# Impact of 6 month conjugated equine estrogen versus estradiol-treatment on biomarkers and enriched gene sets in healthy mammary tissue of non-human primates

**DOI:** 10.1371/journal.pone.0264057

**Published:** 2022-03-17

**Authors:** Gabriel Hobi, J. Mark Cline, Kelly F. Ethun, Cedric Simillion, Irene Keller, Petra Stute

**Affiliations:** 1 Department of Obstetrics and Gynecology, Inselspital, University Hospital and University of Bern, Bern, Switzerland; 2 Department of Pathology, Section of Comparative Medicine, Wake Forest University School of Medicine, Winston-Salem, North Carolina, United States of America; 3 Department of Pathology and Laboratory Medicine, School of Medicine, Emory University, Atlanta, Georgia, United States of America; 4 Biomarkers Core, Yerkes National Primate Research Center, Emory University, Atlanta, Georgia, United States of America; 5 Department of Biology, Interfaculty Bioinformatics Unit, University of Bern, Bern, Switzerland; 6 Department for Biomedical Research, University of Bern, Bern, Switzerland; University of Southern California, UNITED STATES

## Abstract

**Objective:**

To identify distinctly regulated gene markers and enriched gene sets in breast tissue of cynomolgus monkeys (*Macaca fascicularis*) treated for six months with either conjugated equine estrogens (CEE) or estradiol (E2) by analysis of corresponding mRNA levels of genes associated with breast development, carcinogenesis, apoptosis and immune regulation. Additionally, translation of three nuclear markers was analyzed.

**Methods:**

RNA from breast biopsies and necropsies was isolated from two independent study trials from Ethun et al. (CEE) and Foth et al. (E2) after 6 month of treatment duration. RNA was subjected to qRT-PCR and MicroArray analysis. Immunohistochemical stainings were performed for the estrogen receptor alpha subunit (ERa), the progesterone receptor (PGR) and the proliferation marker Ki67.

**Results:**

We identified a total of 36 distinctly enriched gene sets. Thirty-one were found in the CEE treatment group and five were found in the E2 treatment group, with no overlap. Furthermore, two individual genes *IGFBP1* and *SGK493* were upregulated in CEE treated animals. Additional targeted qRT-PCR analysis of ten specific estrogen-related genes showed upregulation of three genes (*TFF1*, *PGR* and *GREB1*) after CEE treatment, respectively one gene (*TFF1*) after E2 treatment. Immunohistochemical stains of breast biopsies showed a significant increase in expression of the PGR marker after CEE treatment.

**Conclusions:**

In this study we identified enriched gene sets possibly induced by CEE or E2 treatment in various processes associated with cancer biology and immunology. This preliminary translational data supports the concept that different estrogen types have different effects on healthy breast tissue and may help generate hypotheses for future research.

## Introduction

Hormone therapy (HT) effectively reduces menopausal symptoms. However, after the first publication of the Women’s Health Initiative (WHI) in 2002 [1] prescription rates of and compliance with HT decreased dramatically worldwide [2]. In the WHI, postmenopausal women with an intact uterus were treated with either conjugated equine estrogens (CEE; 0.625 mg/d) combined with medroxyprogesterone acetate (MPA; 2.5 mg/d) or placebo (WHI-I) while hysterectomized women received either CEE (0.625 mg/d) or placebo (WHI-II), respectively. Interestingly, women treated with estrogen plus progestogen therapy (EPT) were found to have an increased risk of breast cancer (BC) while women who received estrogen therapy (ET) had a sustainably reduced BC risk [3]. Usually, the focus of the discussion about EPT and BC risk lies on the progestogen type [4] while there is uncertainty whether the estrogen type also plays a role. To investigate the impact of estrogen type on healthy mammary gland one would need to collect mammary gland tissue of healthy adult women. However, this is unethical and therefore not feasible. A unique model for the female human mammary gland is the female cynomolgus macaque (*Macaca fascicularis*) mammary gland. From a genetic and endocrine standpoint, female macaques are very similar to women. They possess a more than 95% overall genetic sequence identity to humans, including key genes involved in BC [5]. Furthermore, they have comparable ovarian hormone secretion patterns including a 28-day menstrual cycle and natural ovarian senescence [6, 7]. The human and macaque mammary gland share similarities regarding the microanatomy across development, sex steroid receptor expression profiles, responses to exogenous hormones and the spectrum of developing hyperplastic and neoplastic lesions with aging [8–10]. Due to these unique properties, basic research on estrogen influence on breast tissue of cynomolgus macaques has already been conducted. In a retrospective analysis of nine studies with postmenopausal cynomolgus macaques a more profound impact of E2 on estrogen-induced breast epithelial proliferation than of CEE was established by Wood et al. [11]. The aim of this exploratory study was to identify the impact of estrogen type (CEE, E2) on gene expression profiles in healthy mammary gland of non-human primates.

## Material and methods

### Ethical statement

All procedures using these animals were approved by the Institutional Animal Care and Use Committee (IACUC) of Wake Forest University and were conducted in accordance with federal, state, and institutional guidelines. The facilities and animal resources program of Wake Forest University are fully accredited by the Association for Assessment and Accreditation of Laboratory Animal Care. An ARRIVE (Animal Research: Reporting In Vivo Experiments) Guidelines Checklist has been completed.

### Study design

Randomly chosen raw data from previously analyzed breast biopsies (n = 4 per condition) and randomly chosen breast tissue from previously conducted necropsies was used from two independent previous studies by Ethun et al. [12], respectively Foth et al. [13]. Briefly, the study of Ethun et al. compared effects of 6 month treatment of CEE, bazedoxifene acetate (BZA) and CEE+BZA to control animals [12]. Only previously gathered microarray data and qRT-PCR data from the CEE group and the control group was used in our study for the generation of the presented results. For the immunohistochemical analysis of the CEE group and its control group previously sampled breast biopsies (n = 4 per condition) were stained and analyzed. No data or tissue from animals treated with BZA were used for the work presented here.

The study of Foth et al. compared 6 months of E2 treatment, isoflavone-rich soy protein isolate (SPI) treatment, or both (E2+SPI) to controls [13]. For the work shown here, only samples from E2 treated animals and their respective controls were used. No new animals were held under experimental conditions for the current study. The animal model for both original studies (Ethun et al. and Foth et al.) were colony-born female cynomolgus macaques (*Macaca fascicularis*) [12, 13]. The experimental unit was different groups of animals. As mentioned above, the study of Ethun et al. analyzed the effects of BZA with and without CEE on the breast of postmenopausal monkeys. For the original study of Ethun et al., 100 adult female cynomolgus macaques were imported from the Indonesian Primate Center (Pusat Studi Satwa Primata) at the Institut Pertanian Bogor (IPB) in West Java, Indonesia. Estimated mean age as determined by dentition of all individuals was 12 years. The animals were monoparous or multiparous. Upon arrival at the Wake Forest School of Medicine (WFSM) University Primate Center, all 100 animals were overiectomized and randomized by body weight into social groups (2–5 individuals per social group and per cage). The social groups were then randomly assigned to four experimental groups of n = 25. Due to suspicion of presence of ectopic ovarian tissue (indicated by elevated postovariectomy serum ovarian hormone levels), five animals were excluded. Two excluded animals belonged to the control group and three animals to a BZA-treatment group. No animal of the CEE group was excluded. Treatment was administered in the diet and given once daily. The cake-based diet contained casein, lactalbumin, dextrin, sucrose, cellulose, lard, beef tallow, butter, safflower oil (linoleic), crystalline cholesterol, calcium carbonate, calcium monobasic, minerals and vitamins. The resulting diet composition is shown in S1 Table. The overall time span of the treatment and experiments were from the beginning of 2009 till September 2010. For more information please see the original article of Ethun et al. [12]. The median age for the samples of animals used in the immunohistochemical analysis of this pilot study was 13.1 years for the CEE group and 14.8 years for respective controls. Storage of breast biopsies was continuously at -70° Celsius.

For the study of Foth et al. 56 surgically ovariectomized female animals were used. 43 individuals stemmed from a WFSM breeding colony, which derived from individuals originally imported from the IBP. An additional 13 colony-born individuals were imported from the IPB. All 56 animals were randomized into four treatment groups according to cholesterol days and parity. There were 14 animals per treatment group. Animals lived in social groups of 2–5 individuals. Treatment was administered in the diet and given once daily. The diet of the E2 group and its respective control was also a casein-based cake diet but contained slightly less cholesterol/kcal (S1 Table). Subjective bias was minimized by blinding of the observers to treatment group. During the study, 7 animals were excluded due to gastrointestinal, respiratory, hematologic and unknown pathologies. The overall time span of the experiments were from the end of 1994 until September 1995. Due to an unrelated analysis which examined artery remodeling after injury, the animals were subjected after 3 months of E2 treatment to an aseptic balloon injury of the left iliac artery. All animals were anesthetized during the procedure. Furthermore, after completion of 6 month of E2 treatment and immediately before necropsy (while the animals were already anesthetized), coronary artery reactivity studies were conducted for which the animals were given short-acting endothelium-dependent and independent agonists (acetylcholine & nitroglycerin) [14]. Suspicion for remaining ovary or corpus luteum tissue based on hormonal analysis led to the exclusion of breast necropsy samples of 5 individuals prior to the random selection of tissue for this pilot study. Similarly to the study of Ethun et al. treatment during the original study was administered in the diet and given once daily. Out of the 44 remaining breast samples, 14 belonged to the control group and 13 belonged to the E2 treatment group. Estimated median age of the all individuals per group were 11.2 years in the E2 group and 11.3 years in its respective control group. Out of the remaining stored valid breast necropsies, for the work presented here, 4 of each group were randomly selected for further analysis. The median age for the samples of animals used in this pilot study was 10.5 years in the E2 group and 10.3 years in the control group. Storage of breast necropsies was continuously at -80° Celsius. In the breast necropsies of E2 treated animals analyzed in this pilot study, 3 animals were nulliparous while 1 animal was mono- or multiparous. In the corresponding control group there was 1 nulliparous animal, while the other remaining 3 animals were mono- or multiparous. In addition to records of reproductive history, an assessment of any previous lactation was conducted on the histological appearance of breast tissue by a board-certified pathologist.

In both studies, untreated animals shared similar living and nutritional conditions with the treated. All animals were socially housed in pens with an indoor and outdoor area (each 8x8 square feet). The inside area was made of a masonry construction with epoxy floors and the outside area was made of chain link fencing and concrete floor. Fresh water was freely available in the indoor and outdoor area, as well as perches, play structures and environmental enrichment devices. Biopsies (of CEE treated animals) were harvested under aseptic conditions in a dedicated surgery suite. Animals were sedated with ketamine HCl (15mg/kg intramuscular (IM)) and atropine (0.03 mg/kg IM), tracheally intubated, and maintained on isoflurane anesthesia (1%-2%). Biopsies were performed by an experienced veterinary surgeon. A 2 cm skin incision was made, followed by excision of 0.25–0.5g of mammary tissue. The incision was sutured, and the animals were monitored postoperatively and given analgesia (ketoprofen, 5 mg/kg IM) during recovery following IACUC-approved clinical procedures. Mammary gland was harvested from E2 treated animals immediately after euthanasia in a dedicated necropsy facility. Euthanasia was conducted using ketamine sedation (15 mg/kg) followed by a lethal dose of intravenous sodium pentobarbital (to effect, 50–100 mg/kg). All efforts were made to minimize suffering.

No sample size calculation were made before this pilot study. Rather, due to ethical and economic reasoning, sample size was guided by the amount of already analyzed specimens of the study of Ethun et al. [12]. No experiments involving animals had to be replicated during this study. The animals of the different experimental groups were not assessed in a particular order. No adverse events occurred during this study and therefore no modifications to the protocol were made.

### Microarray analysis

Total RNA was extracted from frozen samples of breast tissue from treated and untreated animals of the E2 trial using TRI Reagent (Sigma Aldrich, St. Louis, MO), purified using a RNeasy Mini kit (Qiagen, Valencia, CA, USA), and quantified using a Nanodrop ND-2000 UV-VIS spectrophotometer (NanoDrop, Wilmington, DE). RNA intactness and quality were confirmed using the Agilent 2100 Bioanalyzer (Agilent, Santa Clara, CA). Only samples with an RNA integrity number (RIN) greater than 7.3 were used for hybridization. 2.5 μg total RNA in ≥20ul nuclease-free water (Qiagen, Valencia, CA, USA) of each sample was subjected to further analysis. Briefly, total RNA was converted into double-stranded complementary DNA (cDNA) using an oligo dT-T7 primer adapter. Double-stranded cDNA was then used as a template for a T7 RNA Polymerase in vitro transcription reaction to produce biotinylated complementary RNA (cRNA) targets. Biotinylated cRNA was fragmented and hybridized to an Affymetrix GeneChip Rhesus Macaque Genome Arrays (Affymetrix, Santa Clara, CA), which were in turn stained, washed and scanned. GeneChip scan files were processed with Expression Console to produce probe set analysis results using the MAS5 algorithm (Affymetrix, Santa Clara, CA). RNA quality control and microarray assays were performed by EA, Quantiles Company (Morrisville, NC).

Breast biopsies of the CEE group and their respective controls were treated similarly in 2012 during the trial of Ethun et al. [12]. In this trial only samples with a RIN greater than 6.0 were used [12]. Raw data gathered during the Ethun et al. trial was used in this exploratory study for the microarray analysis of the CEE group and the respective controls.

### Quantitative RT-PCR analysis

Quantitative real-time reverse transcription polymerase chain reaction (qRT-PCR) was used to measure transcript levels of the following selected markers: Genes associated with cell proliferation (*MKI67* (marker of proliferation Ki67), estrogen receptor activity (*ESR1* (estrogen receptor 1), *ESR2* (estrogen receptor 2), *PGR* (progesterone receptor), *TFF1* (trefoil factor 1), *GREB1* (gene regulated by estrogen in BC-1)), apoptosis (*BCL-2* (B-cell CLL/lymphoma 2)) and estrogen metabolism (*CYP19A1* (cytrochrome P450, family 19, subfamily A, polypeptide), *HSD17B1* (hydroxysteroid 17-beta dehydrogenase 1), *HSD17B2* (hydroxysteroid 17-beta dehydrogenase 2), *STS* (steroid sulfatase, isozyme S), *SULT1E1* (Sulfotransferase family 1E, estrogen-preferring, member 1)). Genes corresponded to those assessed by Ethun et al. [12]. qRT-PCR raw data used for the CEE group and its control was already gathered in 2012 during the Ethun et al. trial. We used qRT-PCR raw data for which corresponding Microarray data was present. This was only for n = 4 per group in the original study. The selection of animals which were subjected to microarray analysis was randomized in the original study [12]. Total RNA was extracted from frozen samples of breast tissue from randomly chosen n = 4 E2 treated and n = 4 control animals of the study of Foth et al. [13] using TRI Reagent (Sigma Aldrich, St. Louis, MO), purified using a RNeasy Mini kit (Qiagen, Valencia, CA, USA), and quantified using a Nanodrop ND-2000 UV-VIS spectrophotometer (NanoDrop, Wilmington, DE). RNA intactness and quality were confirmed using the Agilent 2100 Bioanalyzer (Agilent, Santa Clara, CA). Aliquots of purified total RNA (2μg per sample RNA) were reverse transcribed using the High Capacity cDNA Archive Kit (Applied Biosystems, Foster City, CA) to generate cDNA. Briefly, the master mix (30ul per reaction) for this reaction constituted out of 3ul 10x RT buffer, 1.2ul 25x dNTP, 3ul primers, 1.5ul RT and 6.3ul RNAse free H2O. The RT-PCR was run according to the following protocol: Step 1 (hold) 25°C for 10 minutes. Step 2 (hold) 37°C for 120 minutes. Step 3 (hold) 85°C for 5 seconds. cDNA (9ul) was added to 10ul Taqman Universal Fast Mastermix (Applied Biosystems, Foster City, CA) and 1ul 20x primer probe per reaction. The qRT-PCR was carried out on an Applied Biosystems Fast Real-Time PCR machine (Foster City, CA). *ACTB* and *GAPDH* (glyceraldehyde 3-phosphate dehydrogenase) were used as housekeeping controls. RNA transcript levels were quantified by comparison of relative expression of samples with the control animals. Macaque-specific TaqMan primer-probe assays were used to quantify target transcripts (S2 Table) with normalization to cynomolgus macaque-specific primer-probe sets of housekeeping genes *GAPDH* and *ACTB*. Human probes were used when the sequence was identical. Relative gene expression was determined using the delta-Ct method calculated by ABI Relative Quantification 7500 Software v2.0.1 (Applied Biosystems).

### Immunohistochemistry

Breast tissue sections of the CEE group (biopsies) and E2 group (necropsies) and their controls were sectioned from paraffin blocks at 4 microns, air-dried overnight and then oven-dried for 30 minutes at 60°C. Slides were loaded onto an automated Leica Bond RX machine (Leica Biosystems, Buffalo Grove, IL). The Leica Bond RX machine dewaxed the sections, rinsed and applied citrate buffer (pH: 6) to slides to be stained with antibodies against PGR and ERa. Tris buffer (pH: 8.9) was used for slides which were to be stained with antibodies against Ki67. Sections were then stained using the following commercially available primary monoclonal antibodies for the sex-steroid receptors ERa (NCL-L-ER-6F11, Novocastra Reagents, Leica Biosystems, Buffalo Grove, IL, Dilution: 1:300) and PGR (NCL-PGR, Novocastra Reagents, Leica Biosystems, Buffalo Grove, IL; Dilution: 1:40) and the proliferation marker Ki67 (M7240, Dako Antibodies, Agilent Technologies, Santa Clara, CA, Dilution: 1:800). Incubation duration: 15 minutes. The Bond Polymer Refine Red Detection Kit (Leica Biosystems; Buffalo Grove, IL) was used according to standard protocol. Briefly, a rabbit anti-mouse post primary IgG linker reagent localizes the primary antibodies and is then itself localized via a Poly-Alkaline Phosphates anti-rabbit IgG. This complex is then visualized by precipitate formation after the addition of the substrate chromogen "Fast Red" (incubation duration: 12 minutes). Cell nuclei were counterstained with hematoxylin (incubation duration: 6 minutes). Finally, slides were removed from the instrument and dehydrated using 95% ethanol, 3x100% ethanol and 4x xylene and coverslipped using Permount Mounting Medium (VWR, Radnor, PA). Negative control slides were made with nonimmune mouse serum. Positive control slides were made using known mammary gland tissues if internal markers were not present. IHC slides were scanned using an Olympus VS120 virtual microscopy scanner. To avoid a bias, the counting of the numbers of positively stained cells was done by a blinded examiner. Using the grid function of the ImageJ Program, the virtual slide was divided into even parcels. These parcels were assigned numbers and parcels randomly picked for counting. The computer-assisted manual counting method described by Cline was used to count 100 cells for each slide [15]. A finer grid was placed over the region of interest, and only cells which lay at grid intersections were examined and assigned to the Stained or Unstained category. This was done for markers ERa, PGR and Ki67.

### Statistical analysis

The units of statistical analysis were expression profiles, qRT-PCR data and immunohistochemical data of single animals.

Regarding the microarray data, all raw microarray data was background-corrected, normalized using the RMA method as implemented in the R/Bioconductor package Affy. Probe sets were redefined using the alternative chip definition file "rhesusmamuentrezgcdf", as described by Dai et al. [16]. Differential gene expression was assessed using the moderated t-test as described by Ritchie et al. [17] and implemented in the R/Bioconductor package limma. The Benjamini-Hochberg method was used to perform multiple testing correction. The output of limma was used to perform gene set enrichment analysis (GSEA) using the SetRank method [18]. The key principle of this algorithm is that it discards gene sets that have initially been flagged as significant, if their significance is only due to the overlap with another gene set. It calculates the p-value of a gene set using the ranking of its genes in the ordered list of p-values as calculated by limma [18]. Gene Ontology [19], and KEGG [20] databases were searched for significant gene sets. Enriched gene sets were categorized into six categories based on gene ontology.

All qRT-PCR data were normalised using the mean Ct values of the *ACTB* and *GAPDH* genes. Differential expression was also calculated using the moderated T-test as implemented in the limma package and the Benjamini-Hochberg method was also used here for multiple testing correction. A corrected p-value of 0.05 or less was considered to be significant.

Regarding the immunohistochemical data, identification of pairwise group differences was made with exploratory Mann-Whitney U Test using the MedCalc Software (Version 18.0.0) (MedCalc Software Ltd, Ostend, Belgium). A p-value 0.05 or less was considered to be significant.

## Results

### Animal characteristics

All analyzed tissue derived from ovariectomized animals. In general, animals from the trial of Ethun et al. (CEE) were older (mean age = 14.8 years) than the animals from the trial of Foth et al. (E2). Tissue from the trial of Ethun et al. was stored for a shorter time period and gathered by biopsy instead of necropsy (Table 1) [12, 13].

**Table 1:** Animal characteristics.

Study	CEE [Ethun et al.]		E2 [Foth et al.]	
Treatment group	Control	Treatment	Control	Treatment
Number of animals per group in original study	n = 23	n = 25	n = 15	n = 15
Number of animals analyzed in this pilot study	n = 4	n = 4	n = 4	n = 4
Origin of animals analyzed in this pilot study	Colony-born at IPB	Colony-born at IPB	Colony-born at WFSM	Colony-born: 3 at WFSM 1 at IPB
Dosage (equivalent to women)	nil	0.45 mg/day CEE	nil	1 mg/day E2
Route of treatment administration	nil	in diet, 1x/day	nil	in diet, 1x/day
Diet/Source of protein	Casein/lactalbumin-based cake diet	Casein/lactalbumin-based cake diet	Casein/lactalbumin-based cake diet	Casein/lactalbumin-based cake diet
Cholesterol/kcal	0.29mg/kcal	0.29mg/kcal	0.2mg/kcal	0.2mg/kcal
Estimated mean age as determined by dentition for overall study group	12 years	12 years	11.3 years	11.2 years
Estimated mean age for samples randomly selected for this pilot study as determined by dentition	14.8 years	13.1 years	10.3 years	10.5 years
Parity	Mono-or multiparous	Mono-or multiparous	1 animal nulliparous, 1 animals mono- or multiparous	3 animals nulliparous, 1 animal mono- or multiparous
Ovariectomized	yes	yes	yes	yes
Tissue harvesting after 6 month study duration	Breast biopsy	Breast biopsy	Necropsy	Necropsy
Storage temperature of samples in freezer for microarray analysis/qRT-PCR	-70° Celsius	-70° Celsius	-80° Celsius	-80° Celsius

Abbreviations: CEE = conjugated equine estrogens, E2 = estradiol, IPB = Institut Pertanian Bogor (Indonesia), WFSM = Wake Forest School of Medicine (USA).

### Gene expression profiles

**Fig 1:**
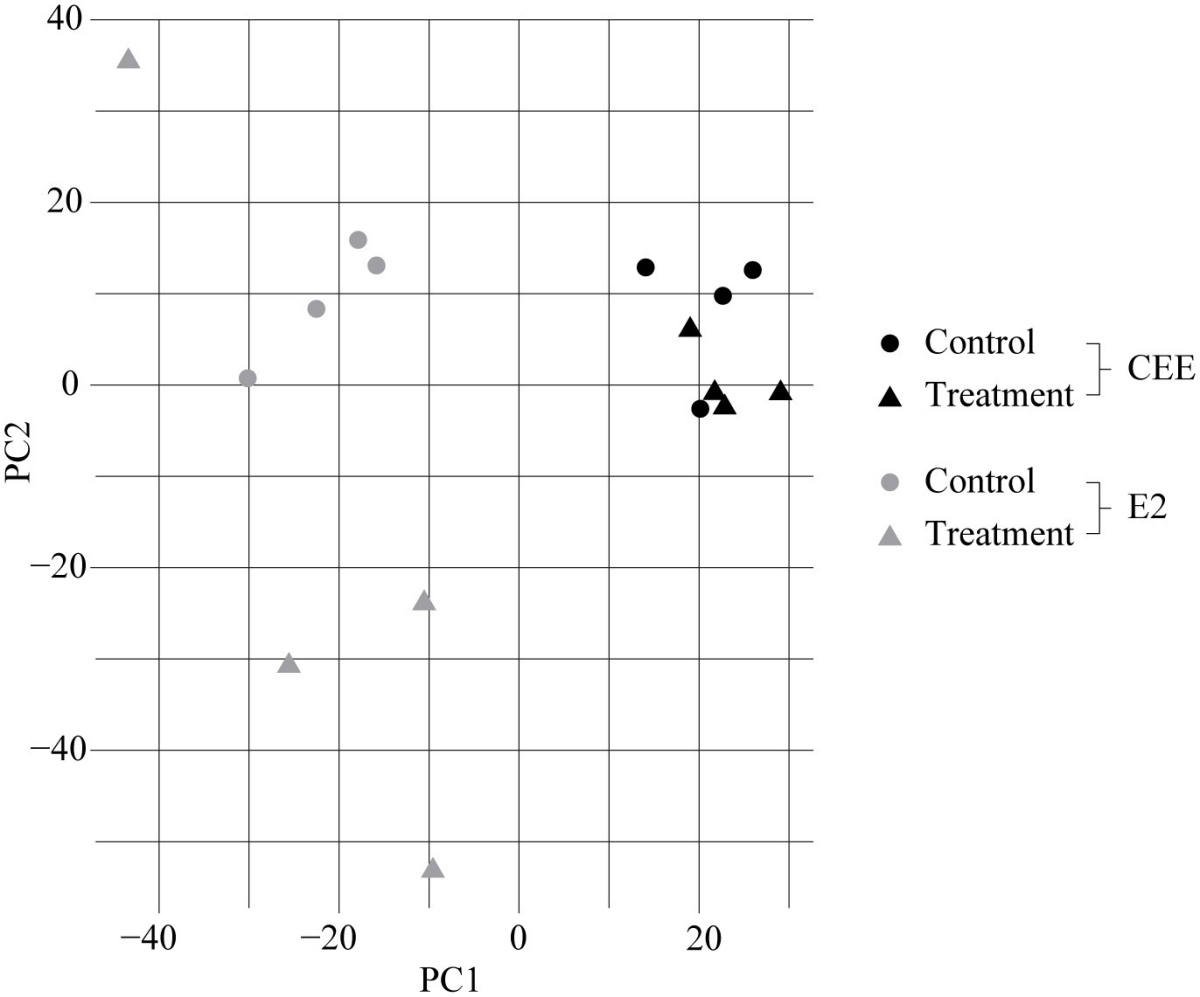
Principal component analysis (PCA) of gene expression profiles in the breast of two treatment groups (CEE or E2) and their respective controls. Abbreviations: CEE = conjugated equine estrogens, E2 = estradiol.

For a gross visualization of the microarray data, dimensionality of the data was reduced by principal component analysis. This algorithm puts a focus on the principal components of each sample, namely the components where the variation is maximal [21, 22]. The principal component analysis (PCA) of our data showed clear separation in mammary gland gene expression profiles between animals originating from the two different studies [12, 13]. Furthermore, divergent vectors were shown for treated (CEE or E2) versus controls among both studies (Fig 1). There was a more distinct separation between E2 treated animals and their respective controls when compared to animals of the CEE study.

### Impact of estrogen type on enriched gene sets

A total of 36 enriched gene sets were identified (adjusted p-value ≤0.05) (Table 2). Comparing CEE treated animals with their controls, 31 enriched gene sets were found (Table 3). These enriched gene sets were associated with the categories immune system, endocrinology, cancer/growth/signaling, bone remodeling, infection/disease and housekeeping/normal function. In contrast, the gene set enrichment analysis of E2 treated animals and their controls yielded only five enriched gene sets. These enriched gene sets were associated with the endocrinology category (Table 4).

**Table 2:** Significantly enriched gene sets (adjusted p-value ≤0.05) as identified by SetRank analysis between CEE vs. controls and E2 vs. controls, respectively.

Annotation	Number of enriched gene sets: CEE vs Control	Number of enriched gene sets: E2 vs Control
Immune System	5	-
Endocrinology	-	5
Cancer/Growth/Signaling	5	-
Bone Remodeling	1	-
Infection/Disease	3	-
Housekeeping/Normal Function	17	-
Total	31	5

Abbreviations: CEE = conjugated equine estrogens, E2 = estradiol.

**Table 3:** Enriched gene sets after CEE treatment *versus* control.

Category	Name	Description	Database	Size	Adjusted P-Value
Immune System	ko04650	Natural killer cell mediated cytotoxicity	KEGG	61	2.76E-05
	ko04933	AGE-RAGE signaling pathway in diabetic complications	KEGG	72	2.76E-05
	ko04623	Cytosolic DNA-sensing pathway	KEGG	39	2.76E-05
	ko04621	NOD-like receptor signaling pathway	KEGG	95	2.76E-05
	ko04660	T cell receptor signaling pathway	KEGG	65	2.76E-05
Cancer/ Growth/ Signaling	ko05203	Viral carcinogenesis	KEGG	121	2.76E-05
	ko05206	MicroRNAs in cancer	KEGG	93	2.76E-05
	ko04350	TGF-beta signaling pathway	KEGG	62	2.75E-05
	ko00562	Inositol phosphate metabolism	KEGG	51	2.76E-05
	GO:0009966	Regulation of signal transduction	GOBP	100	2.76E-05
Bone Remodeling	ko04380	Osteoclast differentiation	KEGG	71	2.76E-05
Infection/ Disease	ko05132	Salmonella infection	KEGG	50	0.0049
	ko05162	Measles	KEGG	80	2.76E-05
	ko05010	Alzheimer’s disease	KEGG	101	0.0051
House-keeping/ Normal Function	GO:0022607	Cellular component assembly	GOBP	94	2.76E-05
	GO:0034645	Cellular macromolecule biosynthetic process	GOBP	104	2.76E-05
	GO:0006351	Transcription, DNA-templated	GOBP	17	2.76E-05
	M00284	Origin recognition complex	KEGG	5	2.76E-05
	ko03010	Ribosome	KEGG	46	2.76E-05
	ko04110	Cell cycle	KEGG	87	2.76E-05
	GO:0016773	Phosphotransferase activity, alcohol group as acceptor	GOMF	43	2.76E-05
	M00285	MCM complex	KEGG	4	2.76E-05
	GO:0006486	Protein glycosylation	GOBP	14	2.76E-05
	M00181	RNA polymerase III, eukaryotes	KEGG	12	2.76E-05
	ko00260	Glycine, serine and threonine metabolism	KEGG	25	0.0003
	ko00280	Valine, leucine and isoleucine degradation	KEGG	32	0.0003
	ko00290	Valine, leucine and isoleucine biosynthesis	KEGG	3	0.0003
	M00147	NADH dehydrogenase (ubiquinone) 1 beta subcomplex	KEGG	8	0.0051
	GO:0045277	Respiratory chain complex IV	GOCC	5	0.0051
	M00143	NADH dehydrogenase (ubiquinone) Fe-S protein/flavoprotein complex, mitochondria	KEGG	7	0.0051
	ko04974	Protein digestion and absorption	KEGG	54	0.0069

Abbreviations: CEE = conjugated equine estrogens, AGE = advanced glycation end products, RAGE = receptor for advanced glycation end products, NOD = nucleotide-binding oligomerization domain, TGF = transforming growth factor, MCM = minichromosome maintenance protein, NADH = nicotinamiddehydrogenase. Size: Number of genes from the reference set that are part of the analyzed gene set. Adjusted P-Value: P-values adjusted for multiple testing using the Holm procedure.

**Table 4:** Differently regulated enriched gene sets after E2 treatment *versus* controls.

Category	Name	Description	Database	Size	Adjusted P-Value
Endo-crinology	ko04012	ErbB signaling pathway	KEGG	57	0.0021
	ko04912	GnRH signaling pathway	KEGG	60	0.0021
	ko04114	Oocyte meiosis	KEGG	76	0.0021
	GO:0007173	Epidermal growth factor receptor signaling pathway	GOBP	5	0.0021
	ko04914	Progesterone-mediated oocyte maturation	KEGG	62	0.0021

Abbreviations: E2 = estradiol, ErbB = Estrogen receptor binding B, GnRH = Gonadotropin releasing hormone. Size: Number of genes from the reference set that are part of the analyzed gene set. Adjusted P-Value: P-values adjusted for multiple testing using the Holm procedure.

### Differences in biomarker gene expression (microarray)

Comparison of CEE treated individuals with controls led to the identification of only two significantly (adj. p-value ≤0.05) differently expressed genes in the microarray analysis (Table 5). In contrast, when comparing E2 treated animals to controls there were no significantly differently regulated genes.

**Table 5:** Genes significantly differently regulated after CEE treatment.

Probe-ID	Entrez-ID	Symbol	Description	logFC	Adjusted P-Value
696994_at	696994	*IGFBP1*	Insulin like growth factor binding protein 1	4.37	0.01
714824_at	714824	*SGK493*	Protein kinase-like protein SKG493	0.94	0.02

Abbreviations: *IGFBP1* = Insulin-like growth factor binding protein 1, *SGK493* = Protein kinase-like protein SGK493. Probe-ID: Refers to the exact probe used in the microarray analysis to identify this gene. Entrez-ID: Unique and stable integer that is assigned to a gene in the Entrez Gene in the National Center for Biotechnology Information’s database for gene-specific information [23]. logFC: log-fold change. Adjusted P-Value: P-value adjusted for multiple testing.

### Differences in biomarker gene expression (qRT-PCR)

**Fig 2:**
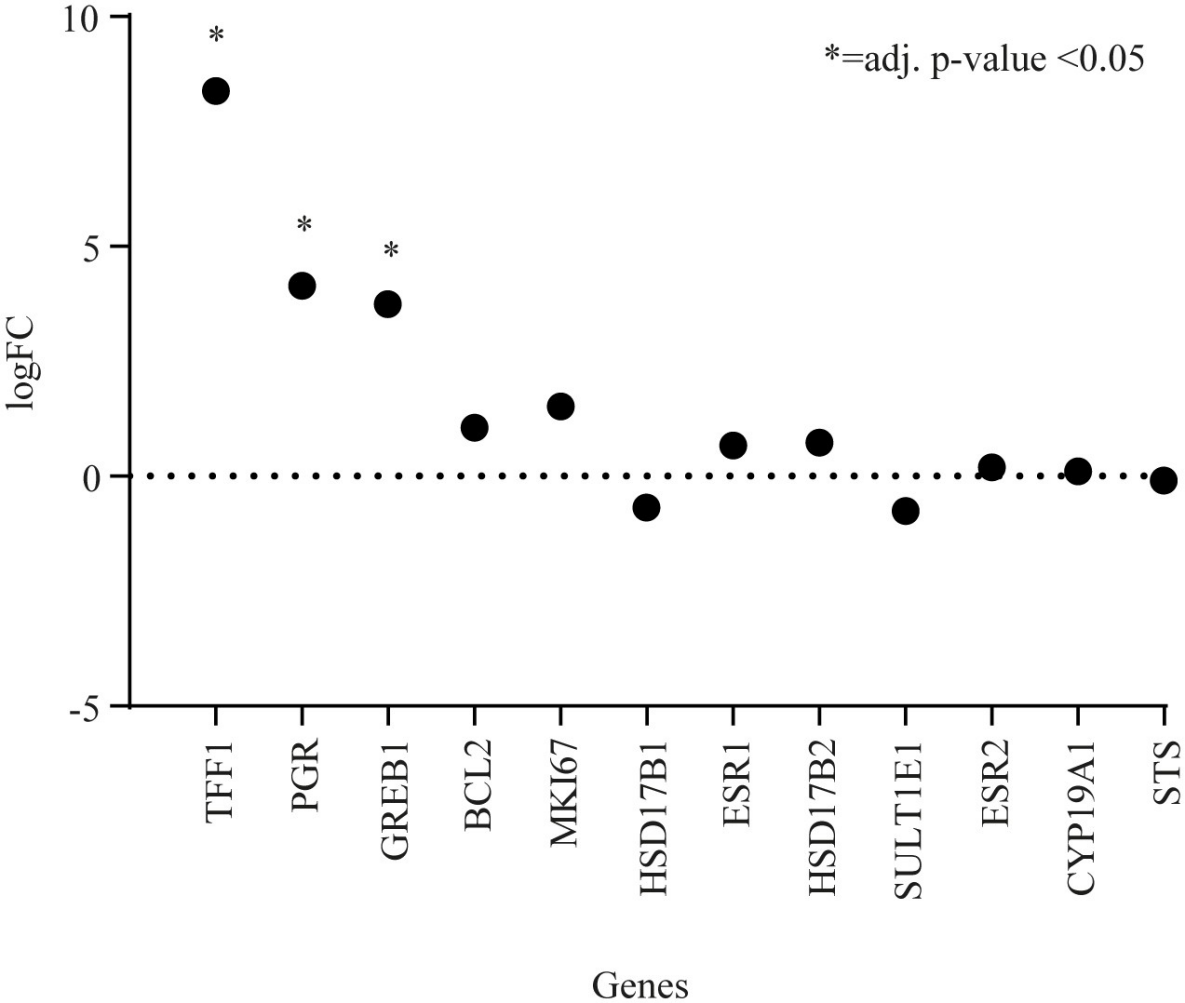
Differences in gene expression by qRT-PCR when comparing CEE treated animals with controls. Abbreviations: qRT-PCR = quantitative reverse transcription polymerase chain reaction, logFC = log base 2 fold change in mRNA expression, *TFF1* = trefoil factor 1, *PGR* = progesterone receptor, *GREB1* = gene regulated by estrogen in breast cancer, *BCL-2* = B-cell CLL/lymphoma-2, *MKI67* = Ki67 Antigen, *HSD17B1* = 17-β hydroxysteroid dehydrogenase [HSD] type 1, *ESR1* = Estrogen receptor 1, *HSD17B2* = 17-β HSD type 2, *SULT1E1* = sulfotransferase family 1E, estrogen-preferring, member 1, *ESR2* = Estrogen receptor 2, *CYP19A1* = Cytochrome P450 Family 19 Subfamily A Member 1, *STS* = estrogen sulfatase.

**Fig 3:**
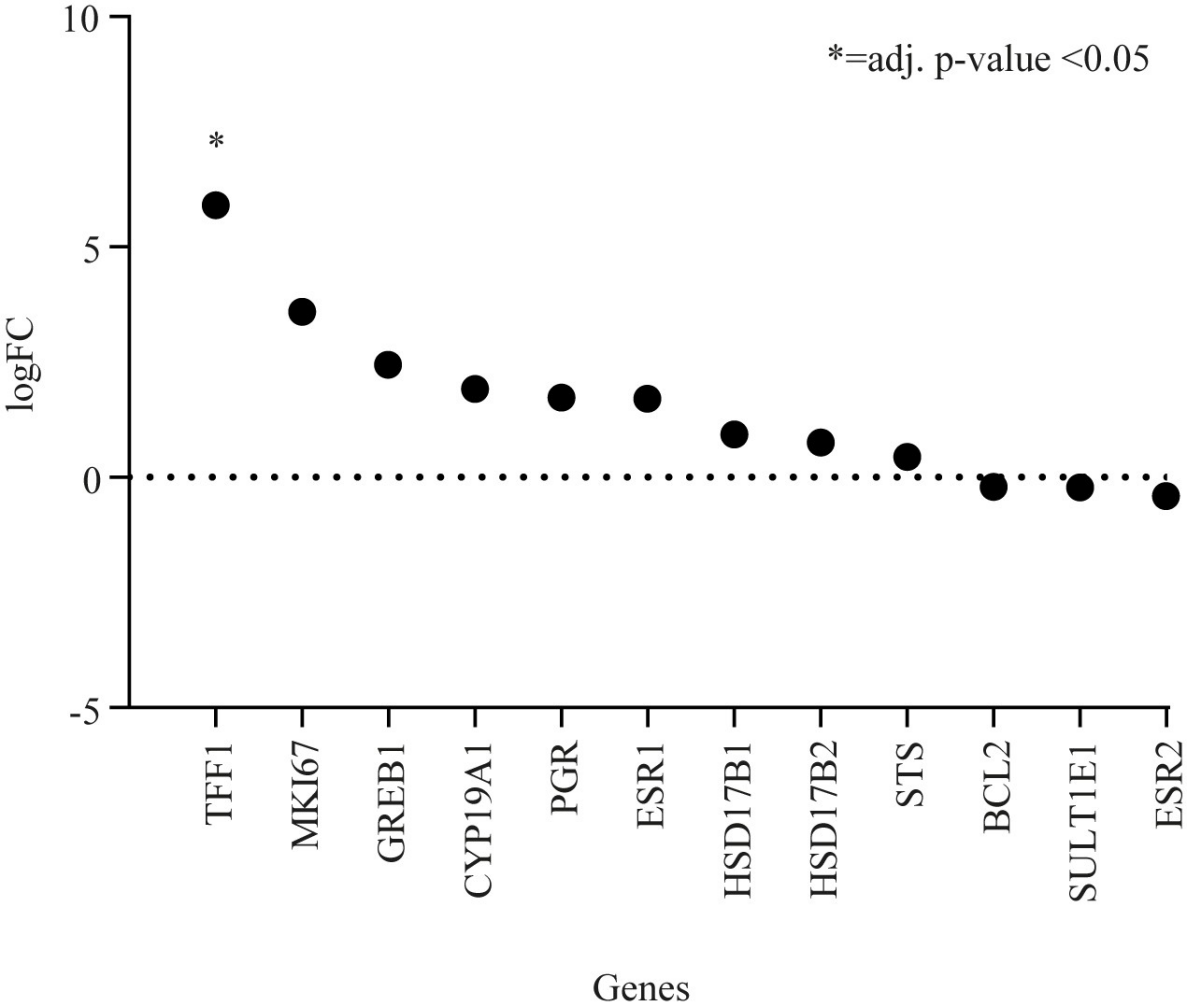
Differences in gene expression by qRT-PCR when comparing E2 treated animals with controls. Abbreviations: qRT-PCR = quantitative reverse transcription polymerase chain reaction, logFC = log base 2 fold change in mRNA expression, *TFF1* = trefoil factor 1, *PGR* = progesterone receptor, *GREB1* = gene regulated by estrogen in breast cancer, *BCL-2* = B-cell CLL/lymphoma-2, *MKI67* = Ki67 Antigen, *HSD17B1* = 17-β hydroxysteroid dehydrogenase [HSD] type 1, *ESR1* = Estrogen receptor 1, *HSD17B2* = 17-β HSD type 2, *SULT1E1* = sulfotransferase family 1E, estrogen-preferring, member 1, ESR2 = Estrogen receptor 2, *CYP19A1* = Cytochrome P450 Family 19 Subfamily A Member 1, *STS* = estrogen sulfatase.

Quantitative RT-PCR was used to assess expression levels of ten key genes previously found to be upregulated by CEE [12]. These genes were associated with breast proliferation (*MKI67*), ER activity (*ESR1*, *ESR2*, *PGR*, *TFF1*, *GREB1*), apoptosis (*BCL-2*) and estrogen metabolism (*CYP19A1*, *HSD17B1*, *HSD17B2*, *STS*, *SULT1E1*). When comparing CEE treated animals with their respective controls only three genes were significantly upregulated in CEE treated animals (Fig 2): *TFF1* (logFC: 8.39; adjusted p-value <0.0001), *PGR* (logFC: 4.14; adjusted p-value <0.0001) and *GREB1* (logFC: 3.75; adjusted p-value <0.0001). When comparing E2 treated animals to their respective controls only *TFF1* was significantly upregulated (logFC: 5.9; adjusted p-value <0.02) (Fig 3).

### Immunohistochemistry of ER, PGR and Ki67 after 6 months of CEE *versus* E2

**Fig 4:**
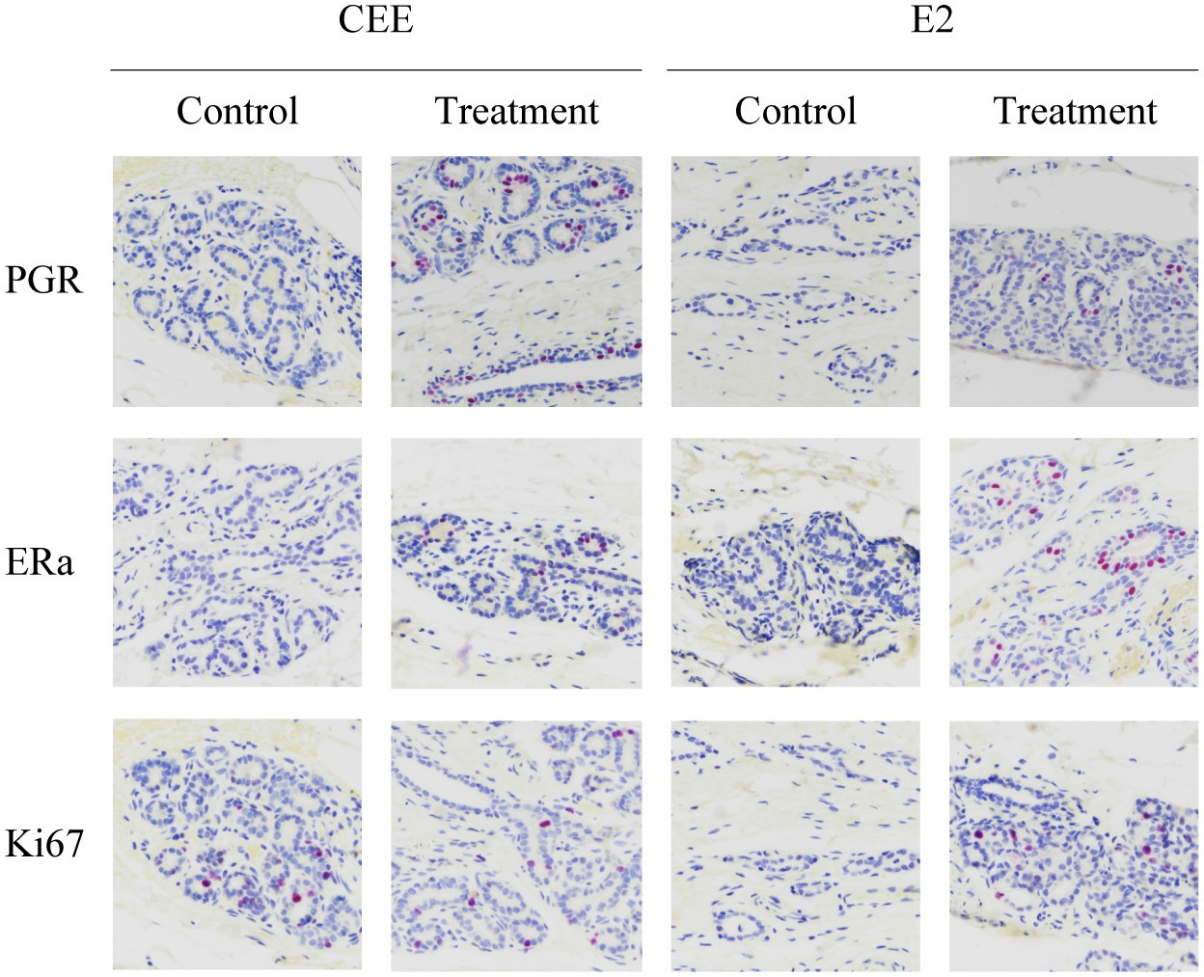
Photomicrographs of *Macaca fascicularis* breast biopsies or necropsies after immunohistochemical staining for nuclear markers PGR, ERa and Ki67. Positively stained cells are purple. Abbreviations: CEE = conjugated equine estrogens, E2 = estradiol, PGR = progesterone receptor, ERa = estrogen receptor alpha, KI67 = Ki67 antigen.

**Fig 5:**
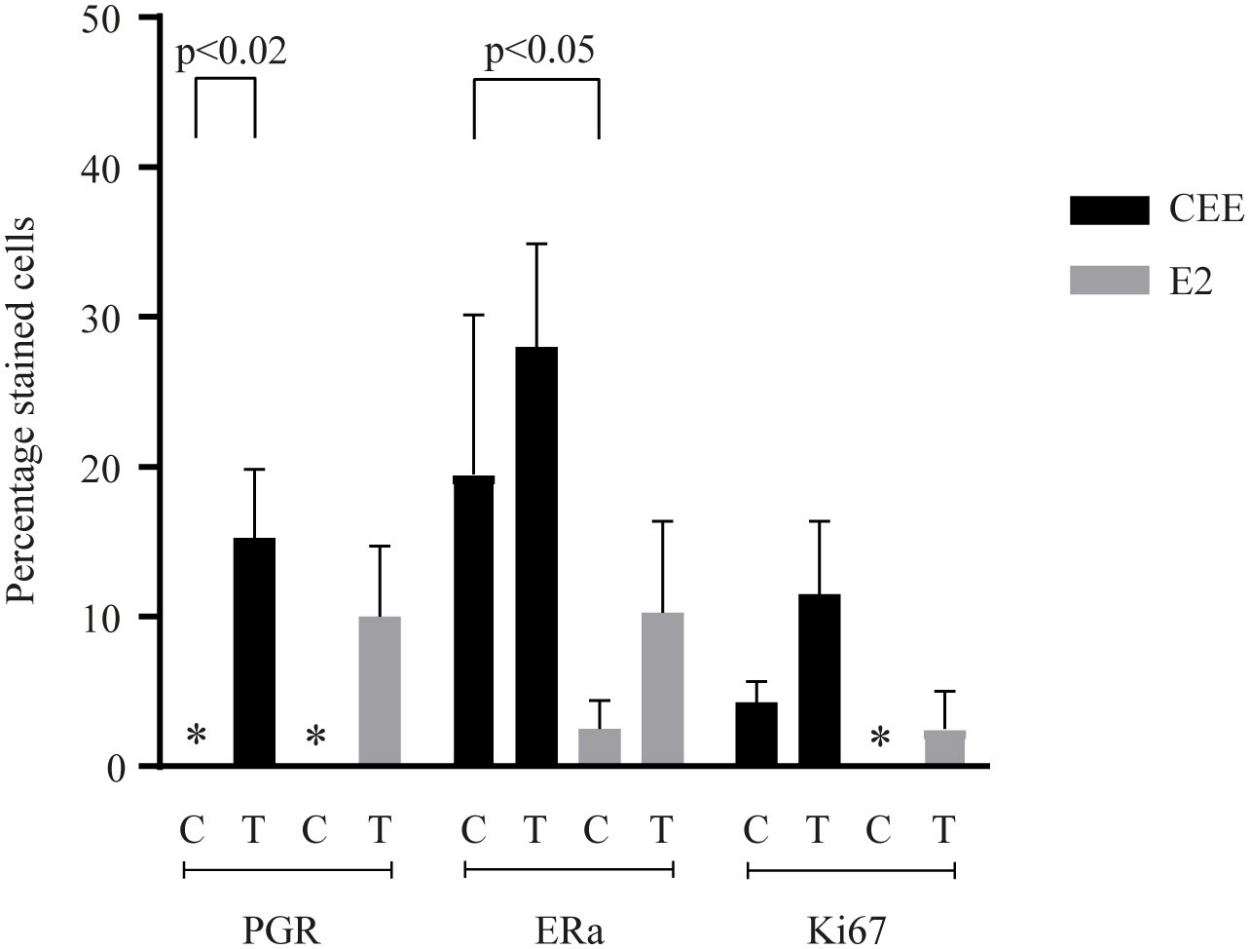
Percentage of stained cells after CEE or E2 treatment and their respective controls. Abbreviations: CEE = conjugated equine estrogen, E2 = estradiol, C = control, T = treatment, PGR = progesterone receptor, ERa = estrogen receptor alpha, KI67 = Ki67 antigen. * indicates true zero. Error bars indicate standard error of the mean.

To evaluate translation of three key genes, immunohistochemistry was performed for ERa, PGR and Ki67. Lobular units were present in all individuals. Representative histologic images of breast tissue for each group are shown (Fig 4). When comparing control animals of both studies ERa protein expression differed significantly. Control animals of the CEE trial had an inherently lower ERa expression (p<0.05) than control animals of the E2 trial. CEE treatment induced a significantly higher PGR protein expression compared to no treatment (p<0.02) (Fig 5).

## Discussion

Regarding the 3Rs of the use of animals in research (replacement, refinement or reduction) according to the ARRIVE (Animal Research: Reporting In Vivo Experiments) Guidelines Checklist [24], no simple optimization step for our pilot study can be proposed since already data and tissue of previously analyzed animals was used. In this study we compared the effects of six months of treatment with CEE versus E2 on the mammary gland of non-human primates. Major findings of the microarray analysis include the identification of 36 distinctly enriched gene sets (CEE n = 31; E2 n = 5). We allocated the identified enriched gene sets to six different categories: immune system, endocrinology, cancer/growth/signaling, bone remodeling, infection/disease and housekeeping/normal function. While enriched gene sets after CEE treatment appeared in all categories except for endocrinology, enriched gene sets after E2 treatment fell all in the category endocrinology.

Regarding the category immune system, all the identified enriched gene sets after CEE treatment were associated with database gene sets regarding early defense against cells undergoing transformation or cellular stress. The found gene sets include the following: ko04650 (Natural killer cell mediated cytotoxicity), ko04933 (AGE-RAGE signaling pathway in diabetic complications), ko04623 (Cytosolic DNA sensing pathway), ko04621 (NOD-like receptor signaling pathway) and ko04660 (T cell receptor signaling pathway). This could reflect a state of increased immune system activation induced by six months of CEE treatment. However, using current data it is impossible to conclude whether this means that CEE treatment could function as a booster for the immune system resulting in a prophylactic protective state or whether these identified gene sets reflect an increase in cellular stress and subsequent activation of the immune system. The expression of the genes associated with the gene sets could combined also have an overall attenuating effect on the immune system. As already noted, there were no enriched gene sets regarding the category immune system distinctly expressed after E2 treatment meaning that E2 treatment could lack a protective immune system boosting effect or leads to less activity of the immune system due to a lower level of cellular stress or transformation. It is important to understand that with current data no comments about directional effects can be made. No enriched gene sets were found after CEE treatment in the category endocrinology. Enriched gene sets which were identified after E2 treatment in this category were as follows: ko04912 (GnRH signaling pathway), GO:0007173 (epidermal growth factor receptor signaling pathway), ko04012 (ErbB signaling pathway), ko04914 (progesterone-mediated oocyte maturation) and ko04114 (oocyte meiosis). The expression of gonadotropin releasing hormone (GnRH) has been found to play a role in many malignant tumours, e.g. cancers of the endometrium, ovary and breast cancer (BC). In malignant tissue GnRH usually has a growth inhibitory effect on tumour cells [25]. It is interesting to notice, that enriched gene sets associated with database gene sets GO:0007173 (epidermal growth factor receptor signaling pathway) and ko04012 (ErbB signaling pathway) were differently regulated after E2 treatment, since intracellular signaling pathways of ErbB and other epidermal growth factor family members have been identified to control proliferation, differentiation and cell cycle progress and are valuable targets in cancer targeted therapy [26, 27]. Enriched gene sets regarding ko04914 (progesterone-mediated oocyte maturation) and ko04114 (oocyte meiosis) are also known to be involved in cell cycle control [28].

In the category cancer/growth/signaling, five enriched gene sets were identified after six months of CEE treatment. These were ko05203 (viral carcinogenesis) and ko05206 (microRNAs in cancer). Both enriched gene sets are associated with various forms of cancer [29, 30]. Furthermore, the following three enriched gene sets were identified: ko04350 (TGF-beta signaling pathway, ko00562 (inositol phosphate metabolism) and GO:0009966 (regulation of signal transduction). TGF beta signaling is a suppressor of epithelial cell proliferation [31]. E2 treatment did not lead to the identification of enriched gene sets in the cancer/growth/signaling category. Further studies are needed to investigate why in the category infection/disease the database gene sets ko05132 (Salmonella infection), ko05162 (Measles) and ko05010 (Alzheimer’s disease) appeared. It is unlikely that individuals of the CEE cohort were suffering from salmonella infection, measles infection or Alzheimer’s disease, but it is possible that genes in these pathways were distinctly regulated by CEE treatment. 17 enriched gene sets were identified in the category housekeeping/normal function in CEE treated animals but not after E2 treatment. This might suggest that CEE also has an effect on breast tissue by altering basic cell function.

In respect to single markers, in CEE treated individuals only two genes were differently expressed. After SetRank analysis they were not associated with any enriched gene set. These were *IGFBP1* (Insulin-like growth factor binding protein 1) and *SGK493* (Protein kinase-like protein SGK493). These genes were already shown to be markedly upregulated in the original study of Ethun et al. among a total of 23 differently expressed genes [12]. The difference between expression levels in this study compared with the original values in the study of Ethun et al. (*IGFBP1* 20.21 fold-change (CEE vs control); *SGK493* 3.37 fold-change (CEE vs control)) albeit using the same data is reflective of the different analysis methods used (ANOVA and pairwise comparison in the original trial of Ethun et al. versus SetRank) and the difference in group sizes for the individual conditions [12]. An association between breast and prostate cancer and I*GFBP1* has previously been reported [32]. SGK493, also known as vertebrate lonesome kinase (VLK), is an extracellular kinase with preference for tyrosine [33] and was shown to regulated cell shape and adhesion in trabecular meshwork cells regarding glaucomas [34]. It is possible that SGK493 also regulates cell shape and adhesion in breast tissue. When comparing the E2 group and their respective controls there was no single gene markedly differentially regulated.

qRT-PCR was done for 10 key genes previously analyzed by Ethun et al. [12]. In the CEE treated animals, albeit using a smaller sample size per treatment than in the original trial, the same three markers, namely *TFF1*, *PGR* and *GREB1*, were differently upregulated in respect to their controls. All three are estrogen-dependent. *TFF1* has been associated with enhancing the oncogenicity of mammary carcinoma cells [35]. PGR controls various functions in the female reproductive tissue and has also been associated with the progression of endocrine-dependent BC [36]. *GREB1*, a target of the transcription factor ERa, is associated with proliferation and regulation of ERa activity in estrogen-responsive BC cells [37]. Hence, the three upregulated gene markers verified after CEE treatment are all associated with BC. Administration of E2 led only to a significant upregulation of TFF1. Lack of the identification of more upregulated genes after E2 treatment using qRT-PCR might likely be due to small group size per treatment making it unlikely for the analyzed gene markers to obtain statistical significance rather than an actual unresponsiveness of the breast tissue towards applied E2 treatment. One E2 treated animal differed markedly from the others in the same treatment group as can be seen in the principal component analysis (Fig 1), making it even more difficult to obtain statistical significance. Since in the previously mentioned retrospective analysis of oral estrogen effects in postmenopausal macaques, standard doses of E2 lead to significant changes of epithelial proliferation in the breast [11], we suspect that more than one out of the 10 analyzed gene markers would show distinct regulation if group sizes per treatment were larger.

By using immunohistochemistry, we were able to analyze three important nuclear markers on a protein level. These were PGR, ERa and Ki67. After six months of CEE treatment, there were significantly more PGR stained cells than in controls. This is in accordance with the qRT-PCR results in which CEE treatment led to an upregulation of PGR gene expression. Furthermore, our results are in accordance with histopathological results of the original study of Ethun et al. in which CEE treatment led to an increase in immunohistochemical expression of PGR in terminal ducts, lobules and extralobular ducts [12]. In contrast to the results of Ethun et al. [12], immunohistochemical expression was not significant for ERa and Ki67. Since biopsies stemmed from the same original study trial, these conflicting results may be due to the method of counting (Ethun et al. subdivided immunohistochemical analysis according to loci) and the smaller group sizes in this study. There was also a significant difference in ERa positively stained cell levels between controls of the two studies. However, since the study setups were independent, this difference was not surprising. Possibly, again due to small sample size, we were not able to show a significant increase of immunohistochemical expression of the proliferation marker of Ki67 like it was previously shown in the original study of Foth et al. [13].

Clearly, our exploratory study has some limitations. For example, as the study populations were inherently different, we were not able to combine control animals of both studies into one group. Although environmental conditions for the cohorts were relatively homogenous within a single study, climate at birth origin, year-specific climate and other environmental changes as well as differences in early nutrition and slight nutritional alterations during the study may have affected our results. The average age of an animal at time of tissue gathering was approximately 3.5 years higher in the study of Ethun than in the study of Foth and gene expression patterns are known to be age-dependent [38]. It is unknown, whether the method of tissue harvesting (biopsy versus necropsy) and the method of sedation has an effect on gene expression. The different lengths (approx. 6 years versus >20 years) and temperatures at which samples were stored (-70° Celsius versus -80° Celsius) might have effects on gene expression. Sufficient quality of stored samples was verified (e.g. by calculation of RIN score) but age-dependent degradation changes cannot be completely ruled out. Furthermore, the use of array analysis in the study of Ethun et al. [12] (CEE) constrained us to use array analysis to examine gene expression in the tissue prepared from the study of Foth et al. [13]. Additionally, due to the intrinsic heterogeneity of the study design, the small number of individuals per group, is a fundamental problem of our study. This exploratory study can only describe qualitatively which genes and enriched gene sets appear and not draw quantitative conclusions. Unlike in the retrospective analysis of Wood et al., where E2 treatment had a more stimulating effect on estrogen-induced breast epithelial proliferation than CEE treatment [11], our current mechanistic data does not allow us to make conclusions about directional effects.

## Conclusion

This exploratory study with its translational medicine approach gives a valuable insight into the effects of six months of treatment with CEE or E2 on the breast tissue of *Macaca fascicularis*. By using the novel SetRank method, genes only slightly differently expressed were clustered and annotated to enriched gene sets, thereby shedding light on genes otherwise overlooked but still possibly involved in the development of breast transformation and BC. The identified enriched gene sets will serve as a starting point for further research and allow for a more focused search for genes of interest. Since found enriched gene sets differ according to treatment, we suspect that a randomized, controlled trial with larger groups is likely to identify differently regulated genes. In conclusion, our exploratory study and its preliminary mechanistic data supports the concept that different estrogen types have different effects on healthy breast tissue and may help generate hypotheses for future research in the quest for more personalized HT strategies.

## Supporting information

S1 Table Diet composition of macaques. (PDF)

S2 Table Macaque primer/probe sets and primer/probe sequences used in qRT-PCR. (PDF)

## Abbreviations

*(M)Ki67*: Marker of Proliferation Ki67
*ACTB*: Actin Beta
AGE: Advanced glycation end products
BC: Breast cancer
*BCL-2*: B-cell CLL/lymphoma-2
BZA: Bazedoxifene acetate (a selective estrogen receptor modulator)
cDNA: Complementary deoxyribonucleic acid
CEE: Conjugated equine estrogens
cRNA: Complementary ribonucleic acid
Ct: Cycle threshold
*CYP19A1*: Cytochrome P450 Family 19 Subfamily A Member 1
E2: Estradiol
EPT: Estrogen plus progestogen therapy
ERa: Estrogen receptor alpha subunit
*ErbB*: Erythroblastic leukemia viral oncogene
*ESR1/ESR2*: Estrogen receptor 1/2
ET: Estrogen therapy
*GAPDH*: Glycerinaldehyde-3-phosphat-dehydrogenase
GnRH: Gonadotropin releasing hormone
*GREB1*: Gene regulated by estrogen in breast cancer 1
GSEA: Gene set enrichment analysis
*HSD17B1*: 17-β hydroxysteroid dehydrogenase type 1
*HSD17B2*: 17-β hydroxysteroid dehydrogenase type 2
HT: Hormone Therapy
IACUC: Institutional Animal Care and Use Committee
*IGFBP1*: Insulin-like growth factor binding protein 1
IHC: Immunohistochemistry
IM: intramuscular
IPB: Institut Pertanian Bogor (Indonesia)
logFC: log base 2 fold change in mRNA expression
MCM: Minichromosome maintenance protein
MPA: Medroxyprogesterone acetate
NADH: Nicotinamide dehydrogenase
NOD: Nucleotide-binding oligomerization domain
*PGR*: Progesterone receptor
qRT-PCR: Quantitative real-time reverse transcription polymerase chain reaction
RAGE: Receptor for advanced glycation end products
RIN: RNA integrity number
RNA: Ribonucleic acid
*SGK493*: Protein-kinase-like protein SGK493
SPI: isoflavone-rich soy protein isolate
*STS*: Steroid sulfatase, isozyme S
*SULT1E1*: Sulfotransferase family 1E, estrogen-preferring, member 1
*TFF1*: Trefoil factor 1
TGF: Transforming growth factor
WFSM: Wake Forest School of Medicine (USA)
WHI: Women’s Health Initiative
