# Supplementary material for: Impact of 6 month conjugated equine estrogen versus estradiol-treatment on biomarkers and enriched gene sets in healthy mammary tissue of non-human primates
Source: PLoS One. 2022 Mar 17;17(3):e0264057. doi: 10.1371/journal.pone.0264057 (PMC8929599; doi:10.1371/journal.pone.0264057)
Supplement: S1 Table — (PDF) [file pone.0264057.s001.pdf]

S1 Table: Diet composition of macaques

Diet/lipid composition	CEE [Ethun et al.]	E2 [Foth et al.]
Carbohydrates (as % of calories)	35.3	42.2
Proteins (as % of calories)	20.3	21.2
Lipids (as % of calories)	44.4	36.6
Saturated fatty acids (as % of total lipids)	45.2	42.9
Monosaturated fatty acids (as % of total lipids)	40.4	37.7
Polyunsaturated fatty acids (as % of total lipids)	14.4	19.4
Cholesterol (mg/kcal)	0.29	0.2
