# Supplementary material for: Impact of 6 month conjugated equine estrogen versus estradiol-treatment on biomarkers and enriched gene sets in healthy mammary tissue of non-human primates
Source: PLoS One. 2022 Mar 17;17(3):e0264057. doi: 10.1371/journal.pone.0264057 (PMC8929599; doi:10.1371/journal.pone.0264057)
Supplement: S2 Table — (PDF) [file pone.0264057.s002.pdf]

S2 Table: Primer/probe sets and primer/probe sequences used in qRT-PCR

**Gene ID**	**NCBI Refseq**	**Assay ID**
BCL2	NM_000633.2	Hs00153350_m1
GREB1	XM_015111718.2	Rh02866842_m1
MKI67	NM002417.4	Hs00606991_m1
PGR	NM_001278457.1	Rh02830094_m1
**Gene ID**	**Accession number**	**Primer/Probe Sequence (5' to 3')[1]**
ACTB	DQ464112	F: ACCCCAAGGCCAACCG<br>R: CCTGGAT**G**GC**C**ACGTACATG<br>P: AAGATGACCCA*ga*TCATG
CYP19A1	DQ529980	F: CCCCAAACCCAATGAATTTACTCTTG<br>R: GGCCCAAAGCCAAATGG**T**T<br>P: AAAGTACCTATAAGGAA*ca*TTCTT
ESR1	DQ469336	F: CACATGATCAACTGGGC**A**AAGA<br>R: TCACATGATCAACTGGGC**A**AAGA<br>P: CACAAAG*cc*TGGCACCC
ESR2	HQ702565	F: GTATCTCTGTGTCAAGGCCATGAT<br>R: CATCCTGGGTCGCTGTGA<br>P: CCTGCTCAATTCCa**a**TATGT
GAPDH	DQ464111	F: TGCCCTCAA**T**GACCACTTTGTC<br>R: ACCCTGTTGCTGTAGCCAAAT<br>P: TCGTTGTCATA*cc*AGGAAAT
STS	DQ529981	F: GGGATGCTGTTGAGGAAATGGA<br>R: CCAATCTCAGCTCATC**T**AGAAGGTT<br>P: ATCTGC*cc*CACACTCC
SULT1E	DQ529982	F: GAGAGAAC**G**GG**C**CAGGTT<br>R: CG**A**GAGGTGTCCTGGATCAG<br>P: ATGTT*cc*TGAGCTCTCG
TFF1	DQ464113	F: GTGCTTCCATCCTAATACCATCGA<br>R: GCAGATCC**A**TGCAGAAGTGTCTAAA<br>P: TCCCTCCAGAA*ga*GGAGTGT
17β-HSD 1	DQ529983	F: CAGGCCTGGGCCT**A**CTG<br>R: C**G**TTCACGTCCAGCACAGA<br>P: CCCA**C**CGCCTCCAGC
17β-HSD 2	DQ529984	F: AAAGG**A**AGGCTGGTGAATGTC<br>R: ATGCCAGC**T**TT**G**CCATTGG<br>P: CCCCT*cc*TCCCATGCTG

NCBI=National Center for Biotechnology Information. [1]Bold letters indicate differences between macaque and human sequences. Italized lowercase of base pairs indicate exon boundaries. Underlined sequence represents an 8-base deletion in the HSD17B1 pseudogene. F, forward primer; R, reverse primer; P, probe.
